# Mucosal affairs: glycosylation and expression changes of gill goblet cells and mucins in a fish–polyopisthocotylidan interaction

**DOI:** 10.3389/fvets.2024.1347707

**Published:** 2024-04-09

**Authors:** Enrique Riera-Ferrer, Raquel Del Pozo, Uxue Muñoz-Berruezo, Oswaldo Palenzuela, Ariadna Sitjà-Bobadilla, Itziar Estensoro, M. Carla Piazzon

**Affiliations:** Fish Pathology Group, Instituto de Acuicultura Torre de la Sal, Consejo Superior de Investigaciones Científicas (IATS, CSIC), Castellón, Spain

**Keywords:** *Sparus aurata*, *Sparicotyle chrysophrii*, aquaculture, parasite, glycosyltransferase, lectin, transcriptomics, mucin sequence

## Abstract

**Introduction:**

Secreted mucins are highly O-glycosylated glycoproteins produced by goblet cells in mucosal epithelia. They constitute the protective viscous gel layer overlying the epithelia and are involved in pathogen recognition, adhesion and expulsion. The gill polyopisthocotylidan ectoparasite *Sparicotyle chrysophrii*, feeds on gilthead seabream (*Sparus aurata*) blood eliciting severe anemia.

**Methods:**

Control unexposed and recipient (R) gill samples of gilthead seabream experimentally infected with *S. chrysophrii* were obtained at six consecutive times (0, 11, 20, 32, 41, and 61 days post-exposure (dpe)). In histological samples, goblet cell numbers and their intensity of lectin labelling was registered. Expression of nine mucin genes (*muc2*, *muc2a*, *muc2b*, *muc5a/c*, *muc4*, *muc13*, *muc18*, *muc19*, *imuc*) and three regulatory factors involved in goblet cell differentiation (*hes1*, *elf3*, *agr2*) was studied by qPCR. In addition, differential expression of glycosyltransferases and glycosidases was analyzed in silico from previously obtained RNAseq datasets of *S. chrysophrii*-infected gilthead seabream gills with two different infection intensities.

**Results and Discussion:**

Increased goblet cell differentiation (up-regulated *elf3* and *agr2*) leading to neutral goblet cell hyperplasia on gill lamellae of R fish gills was found from 32 dpe on, when adult parasite stages were first detected. At this time point, acute increased expression of both secreted (*muc2a*, *muc2b*, *muc5a/c*) and membrane-bound mucins (*imuc*, *muc4*, *muc18*) occurred in R gills. Mucins did not acidify during the course of infection, but their glycosylation pattern varied towards more complex glycoconjugates with sialylated, fucosylated and branched structures, according to lectin labelling and the shift of glycosyltransferase expression patterns. Gilthead seabream gill mucosal response against *S. chrysophrii* involved neutral mucus hypersecretion, which could contribute to worm expulsion and facilitate gas exchange to counterbalance parasite-induced hypoxia. Stress induced by the sparicotylosis condition seems to lead to changes in glycosylation characteristic of more structurally complex mucins.

## Graphical abstract

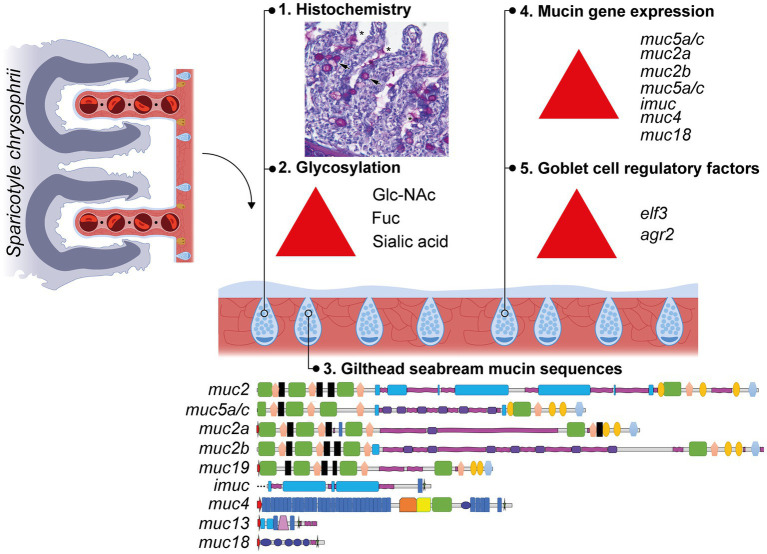

## 1 Introduction

The barrier function of mucosal epithelia is structurally supported by the epithelial cell lining and in a more dynamic manner by the overlying mucus secretion, which is constantly flowing through renewal and off-sloughing. In fish, all exposed body surfaces, including skin, gills, nostrils, and digestive tract, are covered by mucosal epithelia, which constitute first line of defense against external offenders, friction, and dehydration and also participate in disease resistance, respiration, and ionic and osmotic regulation (1, 2). Thus, the role of mucosal health and mucous modulation should not be underestimated in the context of intensive aquaculture rearing systems in a global warming scenario, in which finfish health is under constant threat of imbalance.

The regulation of the mucus-secreting cells and mucus composition upon disease and infection (3–6), including parasitosis (7, 8), is an appealing study topic in human medicine for its diagnostic and therapeutic value (9). Mucus responses in fish skin, gills, and gut have also gathered research interest (10–16). More specifically, gill parasites often provoked mucus over-secretion, as a host mucosal response intended to expel the parasite invader (17–21), but the fish host may have to deal with several drawbacks such as ion and gas exchange imbalance or microbiota dysbiosis.

The flatworm gill ectoparasite *Sparicotyle chrysophrii*, formerly classified in the Monogenea class and recently reclassified into the Polyopisthocotyla class (22), is currently considered the most distressing pathogen for gilthead seabream (*Sparus aurata*) Mediterranean aquaculture (23). The high stocking densities, the lack of fallowing strategies, and the enzootic locations of the off-shore cage farms are favoring parasite outbreaks and transmission. The growing concern in regard to the production losses has directed recent research efforts toward studies on the parasites’ biology (24–28), the host response (29–32), and the search for treatments (33–38). However, a closer look at the modulation of the mucous secretion and the goblet cells in the gills of gilthead seabream during the course of this parasitosis has not been thoroughly taken.

Mucus is mainly produced by goblet cells, secretory cells present in the epithelial cell lining, which synthesize and expel secreted mucins, the main component of the adherent mucus gel. Mucins are high-molecular weight hydrophilic glycoproteins forming a complex matrix, in which water and a cocktail of bioactive molecules are retained, avoiding direct contact between the environment and tissue. By modulating the mucus layer physically (viscosity), biologically (immunoglobulin, lysozyme, antimicrobial peptide contents), and chemically (pH, mucin glycosylation), organisms are able to cope dynamically with the changing external stressors, including their own mucosal microbiota.

The mechanisms that orchestrate goblet cell proliferation, differentiation and distribution patterns, and mucin expression and glycosylation in fish upon parasite challenge are still obscure. This study intends to integrate the study of goblet cell distribution patterns with their transcriptional regulation, together with mucin expression and glycosylation in the gills of gilthead seabream during the course of sparicotylosis using histochemical, lectin-binding, transcriptional, and transcriptomic approaches.

## 2 Materials and methods

### 2.1 Experimental setup and sample collection

Parasite-free, clinically healthy, gilthead seabream juveniles were purchased from a local fish farm and adapted to the indoor experimental facilities of IATS, CSIC under natural photoperiod and temperature conditions (40°5′N; 0°10′E). Along the whole experiment, sea water was 5 μm-filtered and UV-irradiated, salinity was 37.5‰, oxygen saturation was kept above 85%, and unionized ammonia below 0.02 mg·L^−1^. *Sparicotyle chrysophrii* experimental infection was carried out as previously described by Riera-Ferrer et al. (39). In brief, randomly selected recipient fish (R; *n* = 25; initial mean weight 138.40 g ± 22,84 SD) were allocated in a 200 L tank in a recirculating aquaculture system (RAS) connected to a tank with *S. chrysophrii*-infected donor fish. Control unexposed gilthead seabream (C; *n* = 20; initial mean weight 132.30 g ± 11.67 SD) were kept in a separate tank with open water flow, which was disconnected from RAS. The trial lasted for 61 days, and fish were lethally sampled six successive times: 0 days post exposure (dpe) (*n* = 5 C fish); 11 dpe, 20 dpe, 32 dpe, 41 dpe, and 61 dpe (*n* = 3 C fish and *n* = 5 R fish, in each sampling). Fish were culled by tricaine methanesulfonate (MS-222) overexposure (0.1 g·L^−1^) and bled from the caudal vessels and tissue samples obtained for different purposes. The right gill arches of R fish were immediately examined under a stereomicroscope, and intensity and prevalence of *S. chrysophrii* infection were registered. A piece of the left gill arches was fixed in Bouin solution for histological processing, and parallel samples were taken in RNA later for gene expression analysis.

### 2.2 Mucin histochemistry and lectin labeling

Bouin-fixed gills were routinely dehydrated and paraffin-embedded. Sections of 4 μm diameter were stained with periodic acid Schiff (PAS)-alcian blue (pH 2.5), to identify neutral mucins (glycoproteins with oxidizable vicinal diols stained magenta) and acidic mucins (glycoproteins with carboxyl groups and O-sulfate esters stained blue) in all fish samples. Terminal glycoconjugates were analyzed in selected gill sections of 4 C and 4 R fish with medium-high infection intensity (50-115 worms per fish) on 32 dpe. To do so, paraffin sections collected on Superfrost™ Plus slides (Menzel-Gläser, Braunschweig, Germany) were deparaffinized and hydrated; endogenous peroxidase activity quenched in 0.3% hydrogen peroxide for 30 min and incubated for 1 h with six different biotinylated lectins. Following incubation with the avidin–biotin–peroxidase complex (Vector Laboratories, CA, United States) for 30 min, bound peroxidase was finally developed upon a 5-min incubation with 3,3′-diaminobenzidine tetrahydrochloride chromogen (Sigma–Aldrich, MO, United States). The reaction was stopped with deionized water, and the sections were counterstained with Gill’s hematoxylin, dehydrated, and mounted in di-N-butyl-phthalate in xylene. Washing steps between incubations consisted of immersions of 5 min in Tris-buffered saline (TBS, 20 mM Tris–HCl, 0.5 M NaCl, pH 7.2) with and without 0.05% Tween20. Binding specificity of the controls was evaluated by incubating each lectin with its corresponding blocking sugar (0.2 M) for 1 h before the application to the gill sections. Major lectin specificities, lectin sources, blocking sugars and used concentrations are shown in Table 1.

**Table 1:** Lectin sources, specificities, blocking sugars, and concentrations used for labeling.

Acronym	Lectin source	Specificities	Lectin concentration (μg·mL^−1^)	Blocking sugar
Con A	*Concanavalina ensiformis*	Manα-1 > Glcα-1 > GlcAcα-1	2	Methyl-α-D-Man + methyl-α-D-Glc
UEA	*Ulex europaeus*	L-Fucα1,2Galβ1,4	20	L-Fucα
WGA	*Triticum vulgaris*	GlcNAc(β1,4GlcNAc)1–2 > β1,4GlcNAc > NeuNAc	10	GlcNAcα
SBA	*Glycine max*	terminal αβGalNac > αβGal	5	GalNAcα
BSL I	*Griffonia simplicifolia*	D-Gal > D-GalNAc	5	Gal+GalNAcα
SNA	*Sambucus nigra*	NeuAc-α2,6Gal > NeuAcα2,6GalNAc	20	NeuNAc

### 2.3 Microscopic evaluation

Histological staining was performed and analyzed in gill sections of all C and R fish samples. The presence of goblet cell was estimated for the different types of staining with a semiquantitative scoring scale ranging from 0 (absence) to 3 (very abundant, meaning 25–30 cells/microscope field at 500x magnification) at four different gill locations: filament tip, interlamellar pockets, lamellar epithelium, and cartilage-covering epithelium. The intensity of each lectin labeling in these goblet cells was registered according to a semiquantitative scale ranging from 0 (no label) to 3 (very intense label). In addition, the presence and staining of discharged extracellular mucus were registered. Slides were observed under a Leitz Dialux22 (Leica, Hesse, Germany) light microscope, and representative images were taken with an Olympus DP70 Camera (Olympus, Tokyo, Japan).

### 2.4 Mucin gene analyses

The available gilthead seabream genomes [(40) and fSpaAur1.1]^1^ and gilthead seabream sequences in the NCBI database were screened for mucin genes. The obtained sequences that were not previously described in this species (15) were checked by BLAST for verification. The previously available sequences were compared with other sequences in NCBI to further complete partial sequences. The obtained sequences (protein coding regions) were then searched by BLAST against the Ensembl gilthead seabream genome, and the genome location and intron/exon structure were retrieved and represented using online tools.^2^ Protein sequences were analyzed in InterPro^3^ and SMART,^4^ to define protein domains and locations. Primers for new sequences were designed using Primer3 (41). Primer specificity was checked by BLAST against the gilthead seabream genomes, and their efficiency was calculated by serial dilutions (only efficiencies of >90 were considered acceptable).

The sequences of transcription factors relevant to epithelial cell differentiation (goblet cell regulatory factors) were obtained from the NCBI nucleotide database. Primers were designed as described above. Transcript accession numbers and primer sequences used in this study are shown in Table 2.

**Table 2:** Primer sequences used in this study.

Gene name	Symbol	Accession number		Sequence (5′–3′)
Mucin 2a	*muc2a*	XM_030425503	F	ACGCTTCAGCAATCGCACCAT
			R	CCACAACCACACTCCTCCACAT
Mucin 2b	*muc2b*	XM_030414678	F	CCTGTTCAGTGCCCATCCAT
			R	TAAAGCCCAGACTGCAGGTG
Mucin 5 a/c	*muc5ac*	XM_030414679	F	TGGCAATAACACCTGGGGAC
			R	TGTTGTTTGCATGCCACTCG
Mucin 4	*muc4*	XM_030442219	F	GGTGAAGAAGCTGAGGGGTC
			R	TCATTGTACCCAGCCAGCAG
Intestinal mucin	*imuc*	XM_030418634	F	GTGTGACCTCTTCCGTTA
			R	GCAATGACAGCAATGACA
Mucin 18	*muc18*	XM_030399104	F	ATGGAGGACAGAGTGGAGG
			R	CGACACCTTCAGCCGATG
Anterior gradient protein 2	*agr2*	XM_030410519	F	CGACGTTGAGATCCAGAGGG
			R	TCCGGGGAACATACTGTCCA
Transcription factor HES-1-B	*hes1b*	KF857344	F	GAAGCATCTCCGGAACCTCC
			R	GCGGGTGACTTCATTCATGC
ETS-related transcription factor Elf-3	*elf3*	XM_030424933	F	CGAGAAACTAAGTCGGGCGA
			R	TAAACCAGTCTGCGTCCGTC
β actin	*βact*	X89920	F	TCCTGCGGAATCCATGAGA
			R	GACGTCGCACTTCATGATGCT

### 2.5 RNA isolation, cDNA synthesis, and gene expression analyses

RNA from RNAlater^®^ (Thermo Scientific, MA, United States)-fixed gills on 11, 32, and 61 dpe sampling points (3 C and 5 R per sampling) was extracted using MagMAX™-96 total RNA isolation kit (Invitrogen™, CA, United States). RNA concentration and quality were determined using a NanoDrop 2000c (Thermo Scientific, MA, United States), and 500 ng of which was treated with DNase I amplification grade (Invitrogen™, CA, United States). Reverse transcription was performed for 500 ng of input RNA using the High-Capacity cDNA Archive Kit (Applied Biosystems^®^, MA, United States). All procedures were performed following the manufacturer’s instructions.

Real-time quantitative PCR was performed in a CFX96 Connect™ Real-Time PCR Detection System (Bio-Rad). Overall, 20 µl reactions contained 3.3 ng of input cDNA, 5X PyroTaq EvaGreen qPCR Mix Plus (Cultek, Madrid, Spain), and specific primers at a final concentration of 0.45 μM. PCR conditions consisted of an initial denaturation step at 95°C for 3 min, followed by 40 cycles of denaturation for 15 s at 95°C and annealing/extension for 60 s at 60°C. The specificity of the reactions was verified by analysis of melting curves for each reaction. Fluorescence data acquired during the PCR extension phase were normalized by the delta–delta Ct method (42) using *β-actin* as housekeeping gene for normalization, the most stable reference gene in this tissue when compared with other housekeeping genes (*elongation factor 1α*, *α-tubulin*, and *18S rRNA*).

### 2.6 *In silico* analysis of glycosylation enzyme expression

Enzymes regulating glycosylation were screened *in silico*. The gilthead seabream genome (40) was mined for sequences annotated as sialilidases, mannosidases, fucosyltransferases, acetylglucosaminyltransferases, sialyltransferases, mannosyltransferases glucosyltransferases, and phosphomannomutases. Identified sequences were used to mine RNA sequencing results from two previous studies of gilthead seabream infected with *S. chrysophrii*: a study on fish with a mild natural infection [(31); SRA accession PRJNA507368; *n* = 4 control and *n* = 4 infected fish, mean intensity of infection 2.73 parasites/fish] and other on fish with high intensity of infection during an experimental challenge [(43); SRA accession PRJNA992062; *n* = 5 control and *n* = 5 infected fish, mean intensity of infection 121.2 parasites/fish]. Gill-normalized expression values (FPKM) for each identified enzyme were retrieved from the count tables, and differential expression was checked for the DESeq results obtained from each of the studies.

### 2.7 Statistics

Semiquantitative histological scoring on goblet cells and lectin labeling was analyzed among the different infection timings by one-way ANOVA followed by Student–Newman–Keuls using SigmaPlot v14.5 software (Systat Software Inc., CA, United States). When normality or equal-variance failed, the non-parametric Kruskal–Wallis test, followed by Dunn’s post-hoc test for the multiple comparisons, was applied. For all data sets, differences between C and R fish were analyzed by Student’s *t*-test, and when normality failed, the Mann–Whitney *U* sum test was used. Gene expression data were log-transformed (LN) for statistical analyses. For normally distributed data, differences were evaluated using Student’s *t*-test and one-way ANOVA followed by Tukey’s post-hoc test for multiple comparisons. When conditions were not met, non-parametric tests (Mann–Whitney–Wilcoxon or Kruskal–Wallis followed by Dunn’s test) were used. The significance level was set at *p* < 0.05 unless otherwise stated.

## 3 Results

### 3.1 Infection outcome

The prevalence of infection by *S. chrysophrii* was 100%, and detailed data on infection outcome are shown in Table 3. In addition, epitheliocystis, a disease (often secondary) caused by pathogenic intracellular bacteria, was observed in the gills of R fish from 61 dpe on. This bacterial infection provoked the characteristic nodular intracellular inclusions in the gill epithelia.

**Table 3:** Infection outcome in recipient (R) gilthead seabream experimentally exposed to *Sparicotyle chrysophrii*.

	11 dpe		20 dpe		32 dpe		41 dpe		61 dpe	
	Juvenile	Adult	Juvenile	Adult	Juvenile	Adult	Juvenile	Adult	Juvenile	Adult
R (n = 5)	44.4 ± 5.34	0	157.6 ± 14.84	0	0	112.8 ± 43.62	30.40 ± 7.39	46.80 ± 18.08	9.20 ± 5.54	24.80 ± 6.25
T (°C)	17.96 ± 0.13		17.78 ± 0.34		16.03 ± 0.22		14.75 ± 0.07		13.59 ± 0.29	

Mean parasite load of juvenile and adult stages per fish ± SEM and mean temperature (T) ± SEM per each period are given. dpe, days post exposure.

### 3.2 Mucin histochemistry and lectin labeling

**Figure 1:**
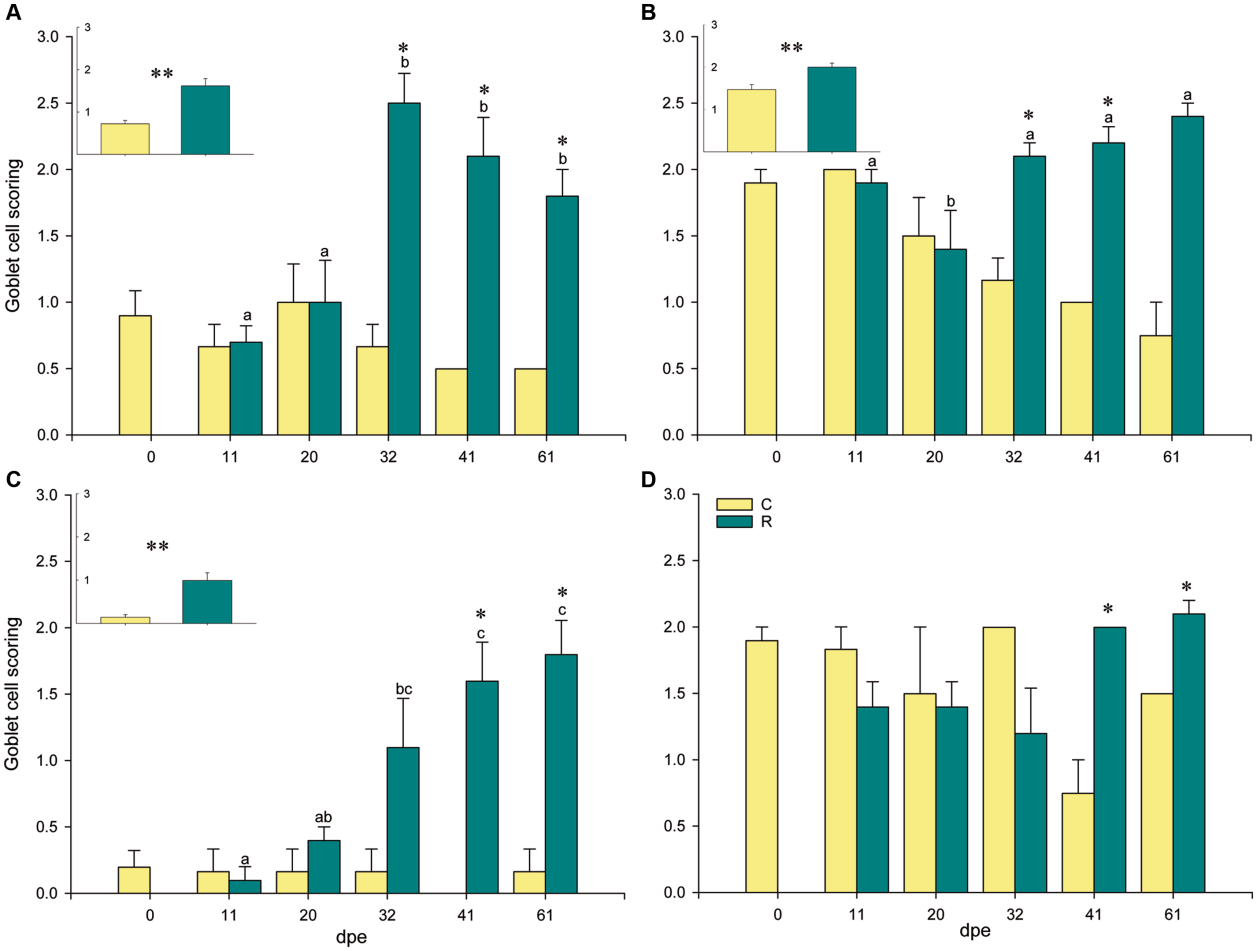
Goblet cell scoring with neutral mucins at different locations in gilthead seabream gills upon *Sparicotyle chrysophrii* infection: interlamellar pocket **(A)**, filament tip **(B)**, lamellar epithelium **(C)**, and epithelium covering the proximal cartilage and adipose tissue **(D)**. Inserts represent pooled data of C or R fish, regardless of the infection timing (*p < 0.05 and **p < 0.001). Different letters stand for statistically significant differences within the R group, *p* < 0.05. Asterisks represent statistically significant differences within the same sampling point (*p* < 0.05). C, control, unexposed fish (*n* = 18); R, recipient, parasitized fish (*n* = 25); dpe, days post exposure.

**Figure 2:**
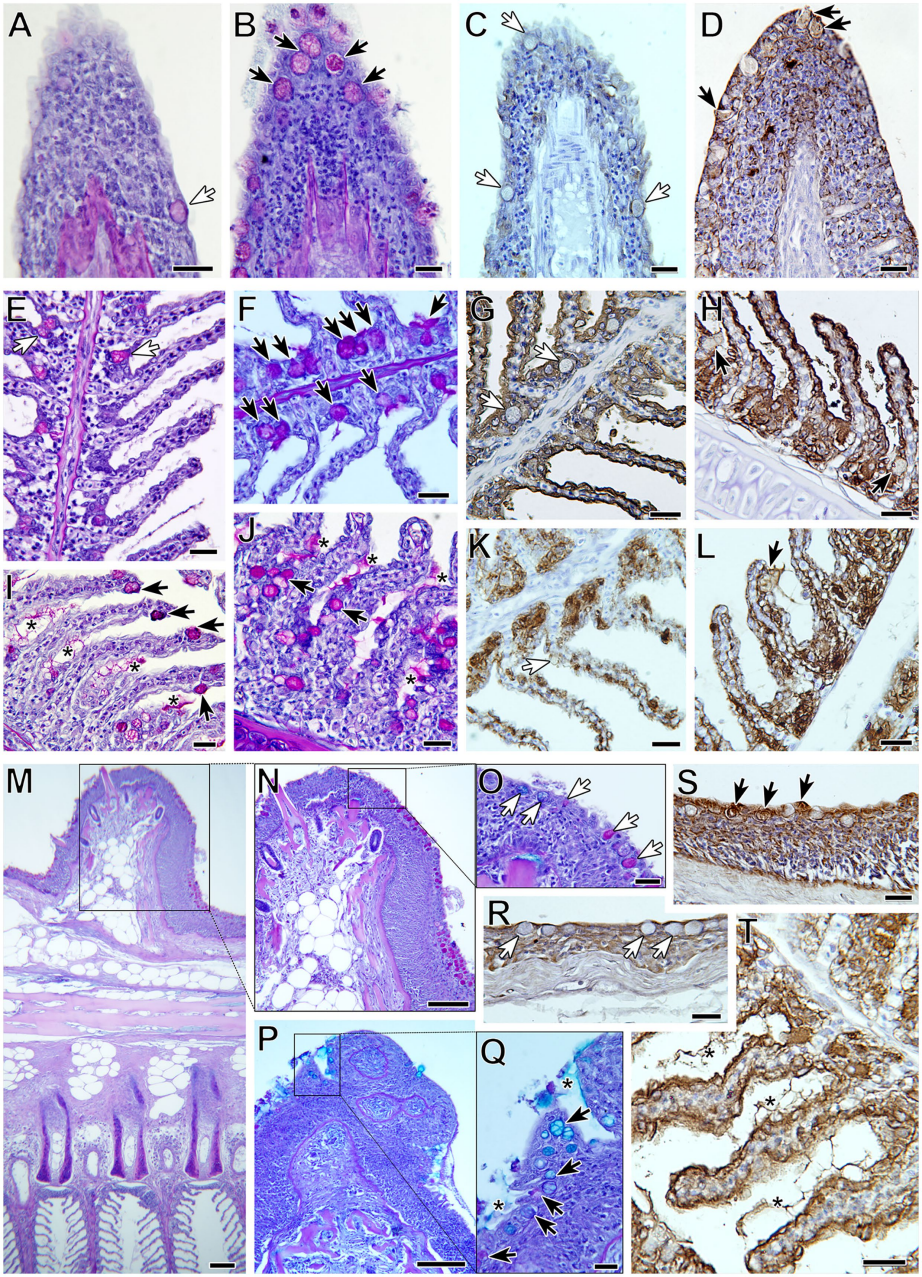
Goblet cell distribution and terminal mucin glycosylation in gilthead seabream gills upon *Sparicotyle chrysophrii* experimental infection **(B,D,F,H,I,J,L,P,Q,S,T)** compared with control gills **(A,C,E,G,K,M,N,O,R)**. Black arrows indicate goblet cells in control gills and white arrows in infected ones. Neutral goblet cell (magenta) hyperplasia was observed in the gills of infected fish at different locations: filament tips **(A,B)**, with increased BSL-I label for Gal **(C,D)**; interlamellar pockets **(E,F)**, with increased WGA label for GlcNac/sialic acid **(G,H)**; along the lamellar epithelium **(I,J)**, with increased SBA label for GalNac **(K,L)**. The epithelium covering the proximal cartilage and adipose tissue **(M)** was the only location with neutral and acidic goblet cells **(N–Q)**, with increased UEA label for Fuc **(R,S)**. Note the presence of interlamellar secreted mucus in infected gills (asterisks), with intense WGA label **(T)**. PAS-alcian blue staining was used in **(A,B,E,F,I,J,M–Q)** and lectin labeling with hematoxylin counterstain in **(C,D,G,H,K,L,R–T)**. All scale bars = 20 μm.

A significant increase in neutral goblet cells was found at the interlamellar pockets, the tips of the gill filaments, and the epithelia of the lamellae, of R fish from 31 dpe on (Figure 1). Neutral goblet cells increased significantly at the epithelium covering the proximal cartilage later, at 41 and 61 dpe, and scoring for acidic and mixed neutral-acidic goblet cells at this gill site was slightly higher in R fish than in C fish along the whole experiment though not significant (Supplementary Figure S1). Goblet cells bearing neutral mucins were ubiquitous in all gill locations, whereas acidic and mixed neutral-acidic goblet cells were only observed at the epithelium covering the proximal cartilage and adipose tissue (Figure 2). Additionally, secreted mucus was often observed in the interlamellar spaces of R fish.

Differences in lectin label intensity among goblet cells of C and R fish were not significant. Nevertheless, some interesting observations were made (Table 4; Figure 2). Lectin label intensity of goblet cells located at the tips of the gill filaments showed an overall decrease, except for BSL I specifically binding to Gal (Figures 2C,D). This terminal sugar was the only one whose staining intensity increased in the goblet cells at all gill locations of R fish. Terminal GalNac residues labeled by SBA also presented an increase in R fish, especially in goblet cells of the lamellae epithelia (Figures 2K,L), but also in the extracellular mucus secretion. Label intensity of terminal GlcNac/sialic acids (WGA) was only higher in goblet cells at the interlamellar pockets of R fish (Figures 2G,H). Fucose sugars evidenced by UEA increased in the goblet cells at all the gill locations of R fish (Figures 2R,S), except at the filament tips. In general, ConA and SNA labels for Man/Glc and NeuNac/sialic acids, respectively, presented the least changes upon parasite infection and mostly decreased in all goblet cells of R fish. However, Man/Glc label was more intense in the interlamellar mucus secretion of R fish.

**Table 4:** Lectin label intensity at the different gill sites in goblet cells (GCs) and in the mucus secretion (Muc sec).

Gill site	Experimental group	Intensity of lectin label					
		ConA	SBA	UEA	WGA	BSL I	SNA
Tip GC	C	3.0 ± 0	2.5 ± 0.5	2.3 ± 0.14	1.5 ± 0	0.0 ± 0	0.1 ± 0.13
	R	2.13 ± 0.72	2.33 ± 0.38	1 ± 0.87	0.83 ± 0.14	1.5 ± 1.06	0 ± 0
ILP GC	C	0.5 ± 0.29	0.5 ± 0.2	0.13 ± 0.13	0.63 ± 0.13	0 ± 0	0 ± 0
	R	0.38 ± 0.38	0.5 ± 0.29	0.25 ± 0.25	0.75 ± 0.14	0.13 ± 0.13	0 ± 0
Epi GC	C	0.38 ± 0.24	0.17 ± 0.14	0.13 ± 0.13	1.25 ± 0.14	0.38 ± 0.13	0.1 ± 0.13
	R	0.25 ± 0.14	1.5 ± 0.2	0.38 ± 0.24	1 ± 0	0.63 ± 0.24	0 ± 0
Prox GC	C	0 ± 0	2 ± 0.2	0.25 ± 0.14	1.63 ± 0.38	0.63 ± 0.13	0 ± 0
	R	0 ± 0	2 ± 0.2	0.63 ± 0.47	1.5 ± 0.2	1 ± 0.2	0.4 ± 0.38
Muc sec	C	0.25 ± 0.14	1.25 ± 0.66	0.63 ± 0.24	1.63 ± 0.8	0.63 ± 0.38	0 ± 0
	R	1 ± 0.29	1.88 ± 0.24	0.5 ± 0.2	2.5 ± 0.35	1.33 ± 0.29	0 ± 0

For each site and lectin, the mean staining intensity ± SEM in control unexposed fish (C, *n* = 4) and recipient parasitized fish (R, *n* = 4) from fish with medium-high infection intensity (50–115 worms in right gill arches) at 32 days post exposure are given. Tip, filament tips; ILP, interlamellar pockets; Epi, lamellar epithelium; Prox, epithelium covering proximal cartilage and adipose tissue. No significant differences were found when comparing C and R groups.

### 3.3 Identification of gilthead seabream mucins

Nine mucin sequences were identified in the gilthead seabream genome, among them five sequences were secreted and four sequences were membrane-bound. The intron–exon structure of the sequences and genomic locations are shown in Supplementary Table S1. Five of these sequences appear complete, beginning with a signal peptide and ending in a stop codon. The structure of the three soluble mucins that do not present a signal peptide seems to indicate that not a large stretch of the sequence is missing. However, the sequence of the membrane-bound *imuc* is clearly incomplete, and although long stretches of repeated mucin domains can be found encoded upstream in the chromosome, the complete structure could not be fully elucidated and will require further sequencing.

**Figure 3:**
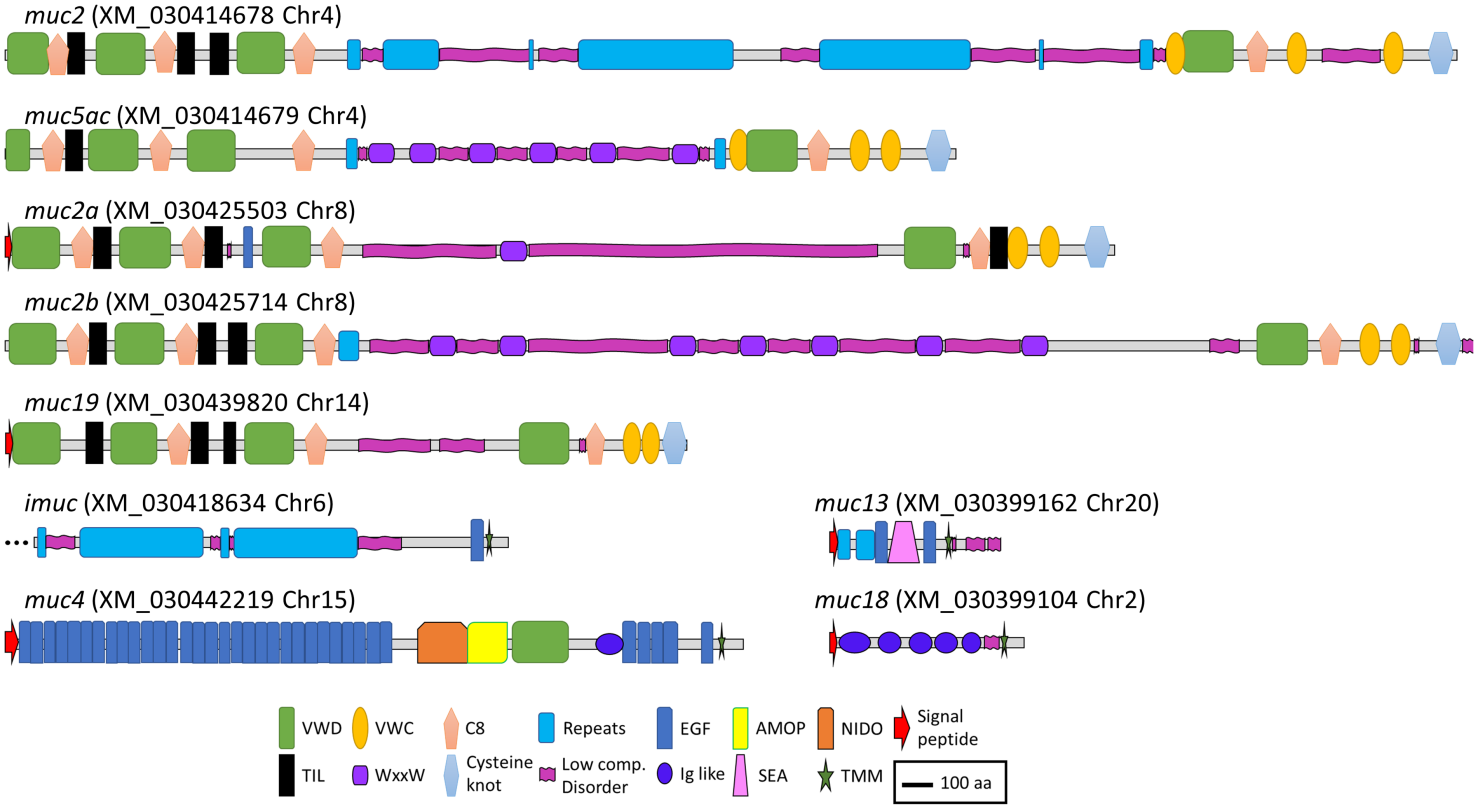
Schematic representation of the deduced amino acid sequences from the mucin genes annotated in the gilthead seabream genome. VWD, Von Willebrand factor type D domain; VWC, Von Willebrand factor type C domain; C8, conserved cysteine-rich domain; Repeats, mucin-like tandem repeats domains; EGF, epidermal growth factor domain; AMOP, adhesion-associated domain in MUC4 and other proteins; NIDO, nidogen-like domain; TIL, trypsin inhibitor-like cysteine-rich domain; WxxW, WxxW domain; Low comp. Disorder, low complexity or disorder domains containing regions rich in the amino acids serine, threonine, and proline (PTS domains); Ig like, immunoglobulin like domains; SEA, sperm protein, enterokinase, and agrin domain; TMM, transmembrane domain. Scale bar: 100 amino acids.

Regarding soluble mucins, in chromosome 4, a sequence annotated as mucin 2 (*muc2*) and another one annotated as mucin 5 ac (*muc5ac*) were identified in tandem, whereas in chromosome 8, two sequences annotated as mucin 2 appeared also in tandem (*muc2a* and *muc2b*). All these sequences showed a similar structure, with three VWD domains and three C8 domains, with a variable number of intercalated TIL domains in the N terminus. The middle of the sequences was characterized by the presence of repeat regions and low complexity or disorder domains that correspond to PTS domains with variable numbers of WxxW domains intercalated. In the C terminus, one VWD and one C8 domain were found in all sequences followed by two VWC domains. All four sequences presented a C terminus cysteine knot domain. A sequence annotated as mucin 19 (*muc19*) was found in chromosome 14, with a very similar structure as the other secreted mucins (Figure 3).

As expected, the structure of the membrane-bound mucins was more variable. The sequence annotated as intestinal mucin (*imuc*), found in chromosome 6, presented a series of repeat domains, followed by an EGF and a transmembrane domain. Mucin 4 (*muc4*), encoded in chromosome 15, showed the characteristic NIDO, AMOP, and VWD domains, followed by several EGF domains before the transmembrane region (44). Mucin 18 (*muc18*), encoded in chromosome 2, showed the typical structure with five immunoglobulin-like domains, a transmembrane region, and a short cytoplasmic tail (45). Finally, a sequence annotated as mucin 13 (*muc13*) was found in chromosome 20, which was characterized by a repeat region followed by a SEA domain surrounded by EGF domains (46) (Figure 3).

### 3.4 Gene expression of mucins and goblet cell regulatory factors

After testing several sets of primers, only six of the nine mucins were found to be expressed in gilthead seabream gills. The two soluble mucins encoded in chromosome 4 (*muc2* and *muc5ac*), *muc2a* from chromosome 8, and the membrane-bound *muc4*, *imuc*, and *muc18*.

**Figure 4:**
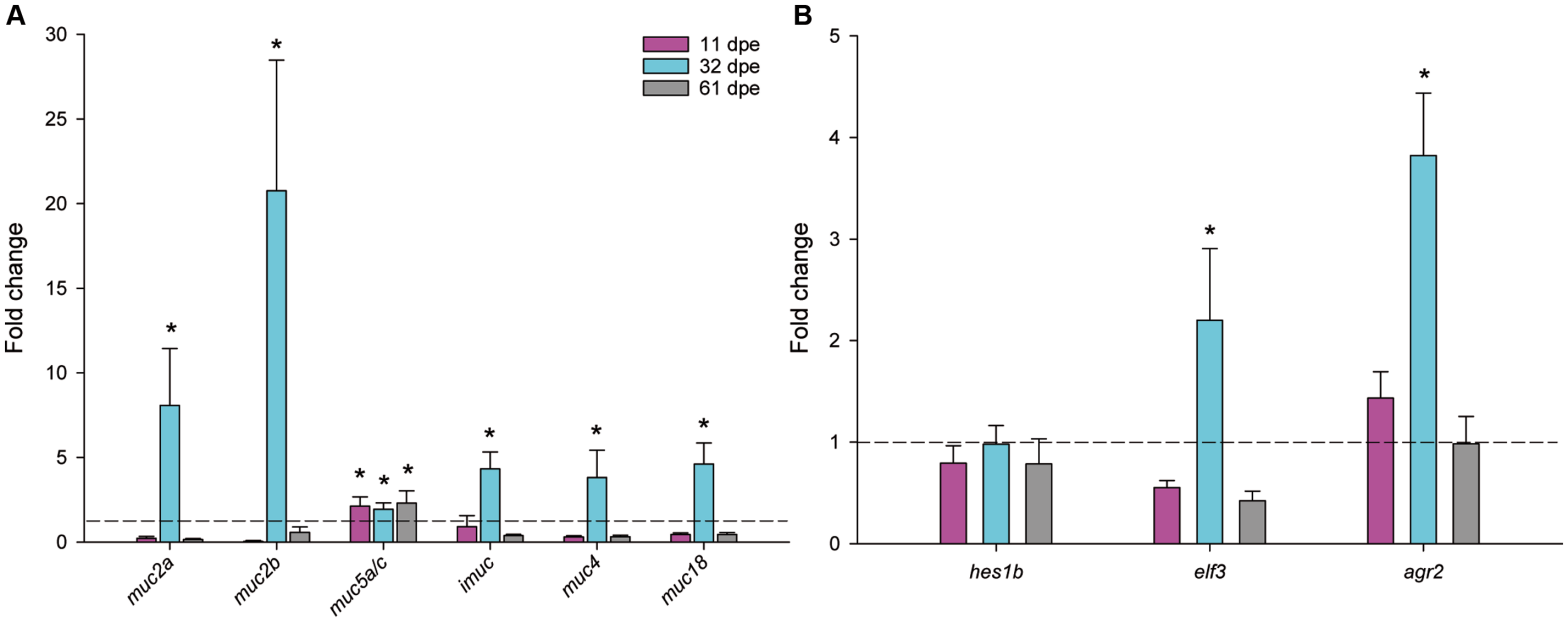
Normalized expression of mucin genes **(A)** and regulatory factors of goblet cell differentiation **(B)** in gilthead seabream gills upon *Sparicotyle chrysophrii* experimental infection (*p < 0.05). Mean fold changes were calculated versus control samples of non-exposed fish ± SEM of *n* = 5. dpe, days post-exposure.

Transcription of mucin genes was robustly upregulated for all detected mucin genes at 32 dpe (Figure 4A). Interestingly, expression of *muc5a/c* was upregulated at all the analyzed timings, and fold changes of the secreted *muc2* and *muc2a* were the highest. The regulatory factors responsible for epithelial cell differentiation *elf* and *agr2* were also significantly upregulated at 32 dpe (Figure 4B).

### 3.5 Genome search for glycosylation enzymes and their differential expression

**Figure 5:**
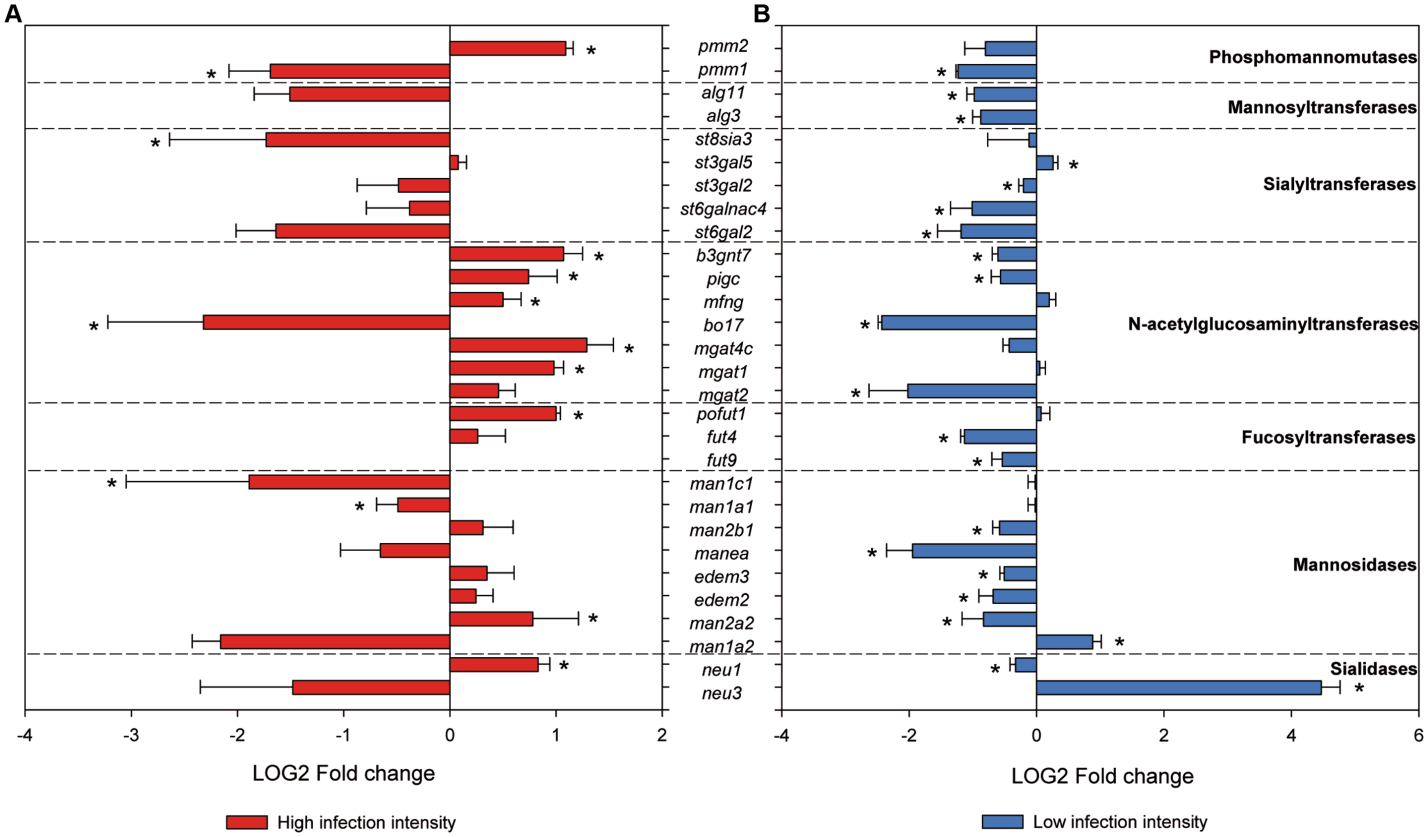
RNAseq normalized gene expression of glycosyltransferases and glycosydases in gilthead seabream gills upon *Sparicotyle chrysophrii* high-intensity experimental infection **(A)** and low-intensity sea cage infection **(B)** (*p < 0.05). Log2 mean fold changes were calculated relative to the control samples of non-infected fish ± SEM of *n* = 5 in **(A)** and *n* = 4 in **(B)**.

Enzymes involved in glycosylation were studied *in silico*. A total of 273 sequences annotated as silialidases, mannosidases, fucosyltransferases, acetylglucosaminyltransferases, sialyltransferases, mannosyltransferases glucosyltransferases, and phosphomannomutases were found in the gilthead seabream genome (Supplementary Table S2A). Examination of the differential expression results from the RNA sequencing experiments by Piazzon et al. (31) and Toxqui-Rodríguez et al. (43) revealed that 29 of these sequences were differentially expressed in at least one of the experiments when comparing control and *S. chrysophrii*-infected fish (Supplementary Table S2B). Marked downregulation was found in fish with low infection intensity (31), whereas fish with high infection intensities (43) presented both significant upregulation and downregulation (Figure 5). Half of these modulated genes responded with opposite trends depending on the infection intensity of the fish, except for the five sialyltransferase genes, which had the same regulation patterns regardless of the parasite load. Higher numbers of significantly regulated genes occurred in low infection-intensity fish than in high infection-intensity fish.

## 4 Discussion

*Sparicotyle chrysophrii* infections in gilthead seabream farms have become a major issue for aquaculture. In the current study, we unveil some aspects of the local mucosal response triggered by this gill ectoparasite, providing valuable data on mucin and goblet cell distribution and regulation. Studies focusing on mucosal immune responses in fish have traditionally focused on the mucosa-associated lymphoid tissues (MALT), their cell effectors, and their molecular signaling. Thereby, the importance of the strictly mucus-related compartment, i.e., the mucus secretion itself, its mucin components, and the responsible goblet cells and goblet cell glycosidases, have been mostly neglected. Nevertheless, in the current-omics era, recent advances in glycomics granted mucus secretion its position as key mediator between epithelial cells, MALT, and microbiota and their joint interactions with external factors such as pathogens (47–49).

The main clinical sign during sparicotylosis is anemia, which is caused by the hematophagous nature of a parasite (32, 39, 50). Furthermore, the parasite’s attaching mechanism to the gill filament through specialized clamps in its opisthaptor region (51) inflicts evident histopathological lesions such as lamellar synechiae and clubbing, resulting in disruption of the epithelium and marginal blood vessels (50). Gill hemorrhages and an increased mucoid exudate are among the recognized pathogenic effects and host response elicited by gill monogeneans, as reviewed by Ogawa (52). Regarding goblet cells, histological scoring of experimentally infected gilthead seabream showed an overall hyperplasia of neutral goblet cells in gill filaments. At the four gill locations where goblet cells were analyzed, a significant increase in neutral goblet cells was detected in R fish compared to C fish at some points during the parasite challenge (Figures 1, 2). This hyperplasia was especially notable and significant over the course of parasite exposure in the interlamellar pockets and the lamellar epithelium from 32 dpe on. A shift in the position of goblet cell distribution toward interlamellar cavities upon infection with the larval parasitic stage of the freshwater mussel *Margaritifera margaritifera* in Atlantic salmon was attributed to their role in gill clearance and remodeling (18). Similarly, the present rearrangement of goblet cell distribution is probably helping, by an increased mucus secretion between lamellae, to lubricate the surface of hyperplasic epithelia avoiding lamellar fusion. Thus, goblet cell hyperplasia was not evident at the other locations away from the lamellae, i.e., filament tips and the epithelium covering the proximal cartilage.

Meaningfully, goblet cell hyperplasia occurred after the first month of parasite challenge once infective oncomiracidia, post-larvae, and juveniles of *S. chrysophrii* turned into the more pathogenic adult worm stages, which was in agreement with the previous observations (39). At 32 dpe, the highest adult stage infection intensity reached 112.8 ± 43.62, with each adult worm bearing 2 rows of up to 72 clamps (personal observation). These, pinch on the lamellae, eroding the mucosal surface and disrupting the gill tissue, probably triggering the increased mucus secretion to protect the nude or damaged gill epithelium. Mucosal and waterborne bacteria infecting such wounds also contribute to an inflammatory response in the parasitized gills (53). In fact, the current *S. chrysophii*-infected fish had developed epitheliocystis at the later sampling point. *Sparicotyle chrysophrii* and epitheliocystis co-infection in gilthead seabream are common under farm and experimental conditions (39, 50, 54), and its specific bacterial etiology was a matter of discussion until recently (43, 55, 56).

In the context of intestinal helminth infections, hyperplasia and hypertrophy of the mucous cells were described at the site of parasite attachment, especially for the ones with acid glycoconjugates (57). Hypertrophy of mucus cells with an increase in carboxylated glycoproteins containing sialic acid was observed in gills of gilthead seabream fed an essential oil-supplemented diet and exposed to *S. chrysophrii*, but no change occurred in cell numbers (34). In the present study, gilthead seabream responded to parasite infection with an increase in goblet cells with neutral glycoconjugates. In healthy gills, the abundant secretion of neutral mucus characterized by glycoproteins with oxidizable vicinal diols is associated with pH regulation for acidity buffering of acidic mucins, with the maintenance of homeostasis and lubrication (58–60). The shift toward mucin acidification upon infection, related to viscosity increase in the mucus secretion and trapping of offending microorganisms due to higher amount of O-sulfate esters (60, 61), was not detected by histochemistry in the gills of *S. chrysophrii*-infected gilthead seabream. Conversely, the predominantly neutral mucin production found from 32 dpe on involves a less viscous mucus layer which facilitates laminar water flow and gas exchange in the gill lamellae (61, 62). Lethargy, as a sign of respiratory distress, is common upon sparicotylosis (38), and even a mild *S. chrysophrii* infection elicited the downregulation of hypoxia-related oxygen homeostasis genes not only locally but also in the spleen of gilthead seabream (31). Thus, the reduced oxygen availability induced by the direct parasitic damage on the gills and blood consumption resulting in anemia provokes severe hypometabolic effects at systemic level during mild infections. Our current results point to a local mucosal response by the fish aiming to counterbalance parasite-induced hypoxia by secreting less viscous mucus on the lamellar surface that facilitates gas exchange.

Goblet cells bearing acidic, carboxylated mucins were found only in the epithelium covering the proximal cartilage of the arches, away from the gill respiratory epithelium, both in C and R fish (Figure 2; Supplementary Figure S1). Rakers and gill arches lubricated with highly viscous and sticky mucus, predominantly containing glycoproteins with O-sulfate esters and sialic acids, were reported from diverse teleost species in order to protect the epithelium of the pharyngeal cavity against mechanical injuries during food ingestion and transport food particles (62). Moreover, glycoproteins in such secretions cross-link sulfate and sialic acid groups resulting in a more resistant barrier against bacterial enzymatic degradation and thus, more difficult to breach by bacteria and more efficient in containing microbial infections (61).

At the transcriptional level, goblet cell hyperplasia was supported by the overexpression of mucin genes and regulatory factors involved in goblet cell differentiation (Figure 4). Apomucin, the linear polypeptide backbone of the mucin, is encoded by specific Muc genes in goblet cells. Assembly, automatic annotation, and *in silico* identification of mucins in non-mammalian species are challenging mainly due to their very long repetitive sequences and the poorly conserved sequence of mucin domains (63). Only a few studies have been conducted on teleost mucin gene expression with mucin transcription data that are barely available for Atlantic salmon (64, 65), zebrafish (66–68), and common carp (69, 70), and one previous record exists for gilthead seabream (15). In the later study, partial sequences and structure of some mucins were described for gilthead seabream from transcriptomic data. In the current study, we integrated this previously defined information with the newly available genome data from two different genome assemblies for this species [fSpaAur1.1 in ensembl.org, NCBI and (40)] and identified nine mucin sequences. Our analysis of the automatic annotation of the sequences showed a coherent complete mucin structure for the membrane-bound mucins (*muc4*, *muc13*, and *muc18*), except for *imuc*, whose C terminus part was the only part identified. However, upstream of the *imuc* sequence in chromosome 6, several large repetitive PST domains can be identified that probably encode a part of the N terminus sequence of this protein, or belong to a different mucin sequence encoded in tandem, as typically found in gel-forming mucin gene organization (71). Long-read sequence analyses should be conducted to unravel the complete sequence of this mucin. Similarly, although *muc2* and *muc5* sequences appear almost complete from N to C terminus, the predicted coding sequences in the middle repeat part are not very consistent, with atypical intron–exon boundary sequences (Supplementary Table S1). In addition, the similarities between *muc2* and *muc5* sequences, and the poor conservation of mucin domains, are challenging for mucin classification and nomenclature in teleosts and other non-mammalian species. In this line, zebrafish *muc5* sequences have been termed *muc5.1–3* instead of using the mammalian nomenclature *MUC5AC*/*MUC5B* (67). Therefore, the nomenclature used in the current study is somewhat arbitrary, and a more accurate annotation of fish mucins should be performed integrating mammalian and non-mammalian sequences, since up to 133 Muc2-type and 263 Muc5-type proteins have been identified from vertebrate genomes (68). Muc2, Muc19, and Muc5 are very large, secreted, gel-forming mucins, whereas I-Muc, Muc4, Muc13, and Muc18 are membrane-bound mucins (15, 72, 73). Pérez-Sánchez et al. (15) found that Muc18, the most abundant gill mucin, and I-Muc constitutively expressed in the gills of gilthead seabream. These authors annotated the *imuc* sequence, which had no clear orthologous genes in mammals and was found highly expressed in the posterior intestine, where it revealed itself as a biomarker of intestinal health changing its expression upon parasite infections. Additionally, expression of *muc2*, *muc13*, and *muc19* was found restricted to the gastrointestinal tract, in agreement with our current results which found no detectable expression in gills. Our results further reveal that I-Muc has an additional role in gill protection and mucosal response (15).

Expression and a robust upregulation of *muc2* genes, *muc5*, *imuc*, *muc4*, and *muc18* were found in the parasitized gills of gilthead seabream at 32 dpe, recovering their initial levels at 61 dpe for all mucin genes except for *muc5a/c*, which remained upregulated along the course of the entire experiment. Apparently, the secreted *muc2* sequences and the membrane-bound *imuc, muc4*, and *muc18* responded acutely once the highly pathogenic adult worms were established, with fold changes up to >20. The highest fold changes among them corresponded to the secreted *muc2a and muc2b*, while upregulation of the membrane-bound mucins *imuc, muc4*, and *muc18* was not as high. Mucin upregulation in response to a gill parasite was described in Atlantic salmon, displaying clinical amoebic gill disease at 21 dpe, when a 30-fold *muc5* upregulation was found (64). In addition, in gills, those authors found downregulation of the membrane-bound *muc18* and very low and variable expression of a *muc2* sequence, which they considered to have a major role in the intestine of salmon. Sveen et al. (65) found three different *muc5a/c* genes, two *muc2* genes, and one *muc5b* gene expressed in the gills of Atlantic salmon, from which two *muc5a/c* and two *muc2* genes were significantly upregulated only 3 h after handling stress. In common carp, different pathogenic viruses provoked severe mucosal distress in gills by downregulating *muc2-like* and *muc18* gene expression at the time that clinical signs of the disease appeared and susceptibility to secondary infections increased (69). In contrast, the expression of three different *muc5* genes was upregulated in the whole body of zebrafish larvae in response to bacterial challenges upon pectin administration, which was considered an innate antimicrobial immune response (66). Therefore, it seems evident that mucin expression is involved in protective responses upon exposure to different pathogens and stressors, but there are large differences in mucin expression patterns among different fish species.

Regulatory mediators are responsible for the tuning of the goblet cell differentiation, leading to their hyperplasia and consequent mucus hypersecretion (74, 75). Accordingly, *elf3* and *agr2*, both involved in goblet cell differentiation, were synchronically upregulated with the observed mucin upregulation in R gills. In the intestines of Elf3-deficient mice, poor differentiation of goblet cells occurred (76). In addition, gene homologs of *agr2* in humans and mice are strongly expressed in mucus-secreting cells of the digestive and respiratory epithelia, and in zebrafish, *agr2* gene expression was found in mucus cells from all mucosal tissues including gills (77). Such regulatory mediators are, however, also responsible for avoiding a chronic inflammatory/repair response, which would worsen respiratory distress and hypoxia (74), and consequently, their expression levels were restored after the second month of parasite exposure. The transcriptional response of *hes1b* was slightly different, presenting a minor downregulation in the gills of R fish along the entire parasite challenge. Nevertheless, in fish as in mammals, the Notch-Hes1 pathway drives intestinal differentiation, and its inhibition results in goblet cell differentiation (78, 79), which is in line with our results. Accordingly, downregulation of *hes1* was described before as an intestinal helminth defense in order to increase mucin secretion and prevent worm attachment or promote detachment (80).

Regarding terminal glycosylation of the mucins contained in gill goblet cells, we found moderate changes including an overall increase in Gal, GalNac, and Fuc in R fish (Table 4). In addition, GlcNac/sialic acid terminal residues also increased slightly in the goblet cells of the interlamellar pockets of R fish. Glycosylation of the apomucin is carried out post-transcriptionally by glycotransferases and glycosidases in the Golgi apparatus during the secretory pathway. The resulting glycan oligomerization and branching and defining of the terminal sugar moieties produce the structural diversity of the glycome. Terminal sugar residues determine the charge and antigens of the mucins and are the molecular basis for interspecies recognition being capable of activating immune responses. In fact, chemical modifications of glycans have been proposed as diagnostic and therapeutic strategies against diseases (81). Firmino et al. (34) suggested that diet supplementation with essential oils during sparicotylosis enhanced the mucosal defense mechanism, in part by an increase in sialic acid-containing mucins, which helped parasite trapping and shedding and led to a significant reduction in parasite abundance and prevalence. Here, in the absence of any particular dietary treatment, WGA binding to almost all sialylated glycans and GlcNAc showed a moderate increase in the goblet cells of the interlamellar pockets and the interlamellar mucus secretion of R fish at 32 dpe. In contrast, SNA, binding only to sialic acid attached to terminal α2, 6Gal, gave the weakest of all lectin labels, being almost negligible. Thus, overall sialylated and Glc-N-acetylated glycans seem to be increasingly secreted in the gills of gilthead seabream as a response to the establishment of adult *S. chrysophrii* stages. The increase in sialic acid and GlcNAc together with goblet cell hyperplasia is recognized as part of the expulsion response against helminth infections, since sialic acids serve for pathogen binding and dumping (8). In agreement, six N-acetylglucosaminyltransferases were upregulated in the RNAseq dataset of gilthead seabream gills with severe sparicotylosis (Figure 5). The addition of GlcNAc to the oligosaccharide chain can generate core 2 and 4 structures, allowing branching and providing the substrate for the further addition of sugar by other glycosyltransferases. This is a regulatory turning point of glycosylation, since many biologically important oligosaccharide structures involved in recognition and adhesion are constructed on this branch, and improper N-acetylglucosaminyltransferase expression in mammals is related to pathological conditions (82). In addition, the expression profile of sialyltransferases was clearly downregulated in the RNAseq dataset of fish with low infection intensity, whereas in highly infected fish, only one sialyltransferase of the five we identified was significantly downregulated, and sialidases presented opposite expression profiles, depending on the infection intensity of the fish.

While sialic acids confer a negative charge to the glycoconjugate, terminal fucose residues confer hydrophobicity, but both are associated with mucosal protection and their alteration may result in disease (72, 75). Thus, human fucosyltransferases and their expression have been studied for their importance in inflammation, mucosal colonization, and host immune response modulation (83, 84), and more recently, their importance for the maintenance of healthy gut microbiota profiles has been stressed out (85, 86). In R gills, a moderate increase in Fuc terminal residues was observed in the lamellar goblet cells and those of the epithelium covering the proximal cartilage, and fucosyltransferase expression appeared upregulated in fish with high infection intensity (Figures 2, 5). Such results may also point to a protective host response through modulation of mucin glycosylation. Conversely, the only previous study including glycosylation of gilthead seabream gills mucus found scarce Fuc residues after feeding with an essential oil-supplemented diet and challenging fish with the polyopisthocotylidan (34). However, those fish had a mean infection intensity of 2.7 parasites/fish, much lower than the current one of 112.8 ± 43.62, and very similar to the 2.73 parasites/fish of the low-intensity RNAseq dataset (31), in which fucosyltransferases were downregulated. Thus, some protective mechanisms of mucosal modulation in the gills of gilthead seabream may only be triggered upon severe polyopisthocotylidan infection. Through a glycomic approach, the presence of complex fucosylated mucin structures was found in Atlantic salmon gills, which would lead to increased structure diversity of glycan epitopes to diversify its repertoire as a possible defensive immune strategy (87). Similarly, the skin mucin structure of Atlantic salmon subjected to chronic stress carried increased Fuc, sialic acid, and core 1 glycans (47).

In addition, GalNAc, which is incorporated by N-acetylgalactosaminyltransferases to the apomucin initiating the oligosaccharide sidechains, seemed to increase in the goblet cells of the R fish lamellar epithelium and the mucus secretion. The detection of this sugar moiety at the terminal position in secreted mucins was considered a sign of immature mucin secretion in gilthead seabream upon intestinal parasite infection (88) and also in vertebrates, in general (89). Secretion of not fully mature mucins seems to contradict the previous observations of mucins with increased GlcNAc, sialic acid, and Fuc, indicators of complex glycans. Future glycomic work will be conducted to validate or reject if the mucosal gill secretion of gilthead seabream contains immature, not fully glycosylated mucins upon sparicotylosis.

Phosphomannomutase isozymes, as known from mammals, are required for the process of N-glycosylation. They provide Man-1-P, the substrate needed by mannosyltransferases, to incorporate Man into glycoconjugates (90). Identified mannosyltransferase sequences participate in this early N-linked glycosylation, from which derived glycan structures are key for functions such as cell recognition, host-defense, and protein secretion in many organisms (91). The downregulation of mannosyltransferase observed in the gills of fish with low infection intensity was not detected in fish with high intensity, as also happened for one phosphomannomutase and most mannosidases identified (Figure 5). Mannosidases and sialidases are both relevant glycosidases for the release of mucin glycans and would play their role in sloughing mucus with the retained parasites off, as previously suggested for an endopeptidase by Firmino et al. (34). Overall, enzyme gene expression from the RNAseq analyses performed herein pointed to a hyporegulative profile in the gills of the fish with low infection intensity, which was mostly reverted or even inverted for many glycosyltransferases and glycosidases in fish with high infection intensity. This would correspond to a defense response in gills involving higher mucin biosynthesis and release, which is in line with the observed goblet cell hyperplasia and mucus hypersecretion upon severe *S. chrysophrii* infection, once the adult parasite stages are established.

Many aspects beyond our current scope, such as glycosyltransferase competition and their intracellular location, epigenetics, health status, or microbiome/pathobiome signaling, can influence the final O-glycosylation profile, which compromises mucin secretion, conformation, involvement in adhesion and recognition events, and microbiome niches. Furthermore, each glycosyltransferase accounts for the proper arrangement of the individual monosaccharides in each unique oligosaccharide structure, and their gene expression serves as fine tuning of the glycosylation process. Future transcriptomic and glycomic approaches will help us understand the scope of the detected mucin modulation in this host–microbiome–parasite interaction by integrating the transcriptional, glycosylation, and microbiome viewpoints. We are still far from understanding the entire interplay occurring between host mediators and effectors, microbiota and microbiota-derived factors, and parasites and their excretory–secretory products. However, this study helps to understand how the gill mucosal microhabitat responds to the *S. chrysophrii* offender and consequent microbiota shift (43) by increased goblet cell differentiation leading to neutral goblet cell hyperplasia on gill lamellae, acutely increased mucin expression, and a probable increase in more complex glycoconjugates with sialylated, fucosylated, and branched structures.

## Data availability statement

The datasets presented in this study can be found in online repositories. The names of the repository/repositories and accession number(s) can be found in the article/Supplementary material.

## Ethics statement

The animal study was approved by the Ethics and Animal Welfare Committee of the Institute of Aquaculture Torre de la Sal (IATS-CSIC, Castellón, Spain) CSIC and “Generalitat Valenciana” (permit number 2018/VSC/PEA/0240). The study was conducted in accordance with the local legislation and institutional requirements.

## Author contributions

ER-F: Formal analysis, Investigation, Methodology, Writing – original draft. RP: Investigation, Writing – original draft. UM-B: Investigation, Writing – review & editing. OP: Funding acquisition, Supervision, Writing – review & editing. AS-B: Funding acquisition, Methodology, Project administration, Resources, Supervision, Writing – review & editing. IE: Conceptualization, Data curation, Formal analysis, Investigation, Methodology, Supervision, Visualization, Writing – original draft, Writing – review & editing. MP: Conceptualization, Data curation, Formal analysis, Funding acquisition, Investigation, Visualization, Writing – review & editing.

## Footnotes

^1^ www.ensembl.org

^2^ wormweb.org/exonintron

^3^ www.ebi.ac.uk/interpro/

^4^ smart.embl-heidelberg.de/
