# Supplementary material for: Mucosal affairs: glycosylation and expression changes of gill goblet cells and mucins in a fish–polyopisthocotylidan interaction
Source: Front Vet Sci. 2024 Apr 9;11:1347707. doi: 10.3389/fvets.2024.1347707 (PMC11035888; doi:10.3389/fvets.2024.1347707)
Supplement: Supplementary file 3 [file Data_Sheet_1.PDF]

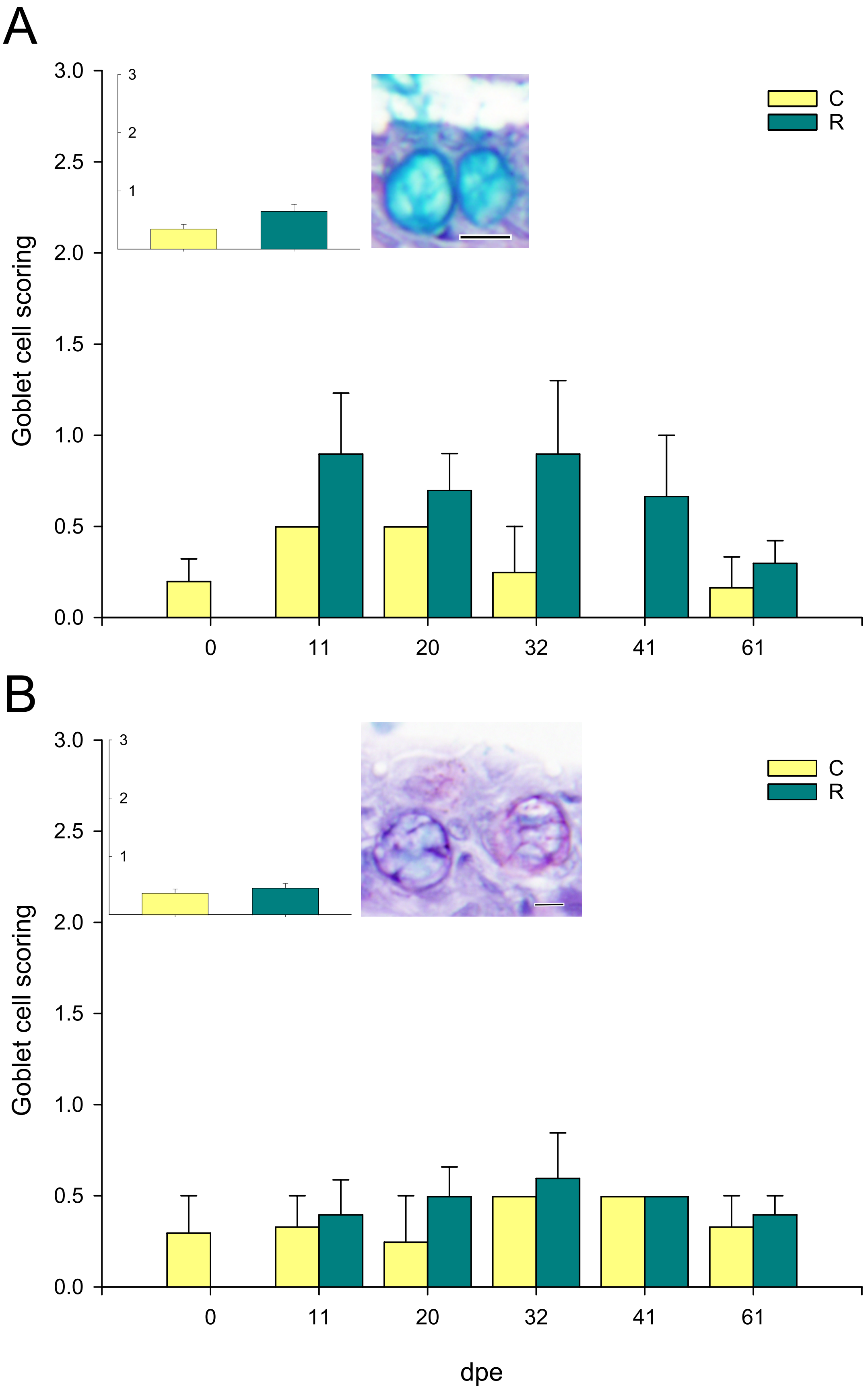

Supplementary Figure 1. Goblet cell scoring with acidic mucins (A) and mixed neutral-acidic mucins (B) in the epithelium covering the proximal gill cartilage of gilthead seabream upon Sparicotyle chrysophrii infection. Graph inserts represent pooled data of C or R fish, regardless of the infection timing; image inserts show the differential staining with PAS-alcian blue. C = control, unexposed fish (n=18); R = recipient, parasitised fish (n=25); dpe = days post exposure. Scale bars = 5 µm.
